# Dynamic Changes in Breast Milk Microbiome in the Early Postpartum Period of Kenyan Women Living with HIV Are Influenced by Antibiotics but Not Antiretrovirals

**DOI:** 10.1128/spectrum.02080-21

**Published:** 2022-04-06

**Authors:** Rabia Maqsood, Peter T. Skidmore, LaRinda A. Holland, Joshua L. Au, Adam K. Khan, Lily I. Wu, Ningxin Ma, Emily R. Begnel, Bhavna H. Chohan, Judith Adhiambo, Grace John-Stewart, James Kiarie, John Kinuthia, Michael H. Chung, Barbra A. Richardson, Jennifer Slyker, Dara A. Lehman, Efrem S. Lim

**Affiliations:** a Center for Fundamental and Applied Microbiomics, Biodesign Institute, Arizona State University, Tempe, Arizona, USA; b College of Health Solutions, Arizona State University, Tempe, Arizona, USA; c School of Life Sciences, Arizona State University, Tempe, Arizona, USA; d Division of Clinical Research, Fred Hutchinson Cancer Research Center, Seattle, Washington, USA; e Department of Biostatistics, University of Washington, Seattle, Washington, USA; f Department of Global Health, University of Washington, Seattle, Washington, USA; g Kenya Medical Research Institute, Nairobi, Kenya; h Department of Paediatrics and Child Health, University of Nairobi, Nairobi, Kenya; i Department of Epidemiology, University of Washington, Seattle, Washington, USA; j Department of Medicine, University of Washington, Seattle, Washington, USA; k Department of Pediatrics, University of Washington, Seattle, Washington, USA; l Department of Research and Programs, Kenyatta National Hospital, Nairobi, Kenya; m Department of Medicine, Emory University, Atlanta, Georgia, USA; n Division of Human Biology, Fred Hutchinson Cancer Research Center, Seattle, Washington, USA; University of Nevada Reno

**Keywords:** antibiotics, breast milk microbiome, combination antiretroviral therapy, HIV, Kenya

## Abstract

Shared bacteria between maternal breast milk and infant stool, infers that transfer of maternal breast milk microbiota through breastfeeding seeds the establishment of the infant gut microbiome. Whether combination antiretroviral therapy (cART) impacts the breast milk microbiota in women living with HIV is unknown. Since current standard of care for people living with HIV includes cART, it has been difficult to evaluate the impact of cART on the microbiome. Here, we performed a next-generation sequencing retrospective study from pre-ART era clinical trials in Nairobi, Kenya (between 2003–2006 before cART was standard of care) that tested the effects of ART regimens to prevent mother-to-child HIV transmission. Kenyan women living with HIV were randomized to receive either no ART during breastfeeding (*n* = 24) or cART (zidovudine, nevirapine, lamivudine; *n* = 25) postpartum. Using linear mixed-effects models, we found that alpha diversity and beta diversity of the breast milk bacterial microbiome changed significantly over time during the first 4 weeks postpartum (alpha diversity *P* < 0.0007; beta diversity *P* = 0.005). There was no statistically significant difference in diversity, richness, and composition of the bacterial microbiome between cART-exposed and cART-unexposed women. In contrast, antibiotic use influenced the change of beta diversity of the bacterial microbiome over time. Our results indicate that while early postpartum time predicts breast milk microbiome composition, cART does not substantially alter the breast milk microbiota in women living with HIV. Hence, cART has minimal impact on the breast milk microbiome compared to antibiotics use.

**IMPORTANCE** Breastfeeding has important benefits for long-term infant health, particularly in establishing and shaping the infant gut microbiome. However, the impact of combination antiretroviral therapy exposure and antibiotics on the breast milk microbiome in women living with HIV is not known. Here, in a longitudinal retrospective study of Kenyan women living with HIV from the pre-antiretroviral therapy era, we found that antibiotic use significantly influenced breast milk microbiome beta diversity, but antiretrovirals exposure did not substantially alter the microbiome. Given the protective role of breastfeeding in maternal-infant health, these findings fill an important knowledge gap of the impact of combination antiretroviral therapy on the microbiome of women living with HIV.

## INTRODUCTION

Breast milk provides protection and health benefits to infants due to its unique composition of nutrition, microbiota, antibodies, and human milk oligosaccharides (HMOs) (1–3). Women living with HIV (WLHIV) in sub-Saharan Africa are encouraged to exclusively breastfeed their babies for the first 6 months postpartum while on combination antiretroviral therapy (cART) (4) to increase infant survival by reducing exposure and providing immune protection against pathogens (1).

Previous studies have characterized the breast milk bacterial microbiome and found a core microbiome in breast milk from women across geographic regions including Kenya, Spain, and the United States, among others (5–7). *Staphylococcaceae and Streptococcaceae* are the most abundant and prominent bacterial families found in breast milk universally. Studies have also shown shared bacterial taxa between maternal breast milk and infant stool, suggesting that breast milk seeds and populates the infant gut bacterial microbiome (3, 8, 9). Therefore, disruptions in the composition of breast milk may cause alterations of infant gut bacterial microbiome, which has previously been linked to a wide array of chronic disorders such as Crohn’s disease (CD), diabetes mellitus, and obesity (10–13). The gut microbes are thought to play a role in immune-mediated or inflammatory processes that lead to these disorders (14).

Previous studies have shown that among WLHIV who are not on ART, the core breast milk bacterial microbiome is similar to that of HIV-negative women (5, 6, 15), and that immunosuppression does not appear to have a substantial impact on either breast milk DNA virome or bacterial microbiome (5). However, data is lacking on the impact of ART on the breast milk bacterial microbiome. Because studies have shown that ART can impact gut bacterial microbiome independent of HIV infection (16–18), we hypothesized that cART could alter the breast milk bacterial microbiome. This could impact infant health as changes to the breast milk bacterial microbiome could alter the transfer of beneficial or pathogenic species or alter immune responses or inflammation. We conducted a retrospective study using specimens from historic prevention of mother-to-child-transmission (PMTCT) trials to determine whether cART changed the dynamics of the bacterial microbiome in breast milk during the first month postpartum in Kenyan WLHIV.

## RESULTS

### Study population.

This study is a retrospective analysis of breast milk samples collected between 2003 and 2006 from WLHIV participating in two randomized trials evaluating PMTCT regimens in Nairobi, Kenya (19–21) (see Materials and Methods). The trials evaluated contemporaneous PMTCT regimens prior to the widespread rollout of cART in Kenya, and details of recruitment, enrollment, and follow-up have been described in detail elsewhere (19–21). As was the standard of care at the time of the original studies, one cohort of mothers was given short-course zidovudine (ZDV) during their last trimester of pregnancy to reduce the risk of mother-to-child transmission (19). Given the short half-life of ZDV (1–2 h), this group of mothers act effectively as the cART-unexposed group for this study after the first 2 days postpartum (21). A second cohort of women was randomized to cART who were given ZDV/NVP/3TC during pregnancy and 6 months postpartum (20) and serve as the cART-exposed group in this study.

We found no significant differences between the cART-exposed and unexposed treatment groups in terms of age, parity, premature labor, or mastitis (Table 1). However, due to differences in inclusion criteria of the original trials, cART-exposed had lower CD4 counts (collected during pregnancy) than the cART-unexposed women and had significantly lower median HIV RNA and DNA levels in breast milk (21). cART-exposed women were more likely to have taken folate and iron supplements during pregnancy, while antibiotic use was higher in cART-unexposed women, although the difference was not statistically significant (Table 1).

**TABLE 1:** Population Characteristics

Maternal characteristics	No cART (*n* = 24)	cART (*n* = 25)	*P* value
Maternal age (median, IQR, range)	25.5 (21.875, 29.25), 18 to 39	26 (24, 29), 18 to 34	ns^*a*^ (0.989)
Parity (median, IQR, range)	2 (0.5, 2), 0 to 5	1 (1, 2), 0 to 3	ns (0.542)
Primiparous, no. (%)	18 (75%)	20 (80%)	ns (0.742)
Antenatal/delivery characteristics			
Premature labor, no. (%)	4 (16.7%)	1 (4%)	ns (0.190)
Baseline CD4 count (median, IQR, range)	446 (268, 660), 155 to 1507	280 (248, 421), 200 to 481	0.049
Baseline CD4 percent (median, IQR, range)	24 ( 6.5, 29.5), 6 to 48	24 (19, 29), 13 to 29	ns (>0.999)
Plasma HIV RNA (median, IQR, range)	4.6 (4.4, 5.4), 4 to 6	4.9 (4.4, 5), 2.9 to 6.3	ns (0.984)
Postpartum characteristics			
Mastitis (ever during first mo), No. (%)	4 (16.7%)	3 (12%)	ns (0.702)
Breast abscess (ever during first mo), no. (%)	1 (4.2%)	2 (8%)	ns (>0.999)
Supplements/medications			
Iron, no. (%)	7 (29.2%)	17 (68%)	0.010
Folate, no. (%)	8 (33.3%)	16 (64%)	0.047
Any antibiotic usage, no. (%)	10 (41.7%)	5 (20%)	ns (0.128)

a ns, not significant.

### Longitudinal dynamics and correlates of breast milk bacterial microbiome diversity.

**FIG 1:**
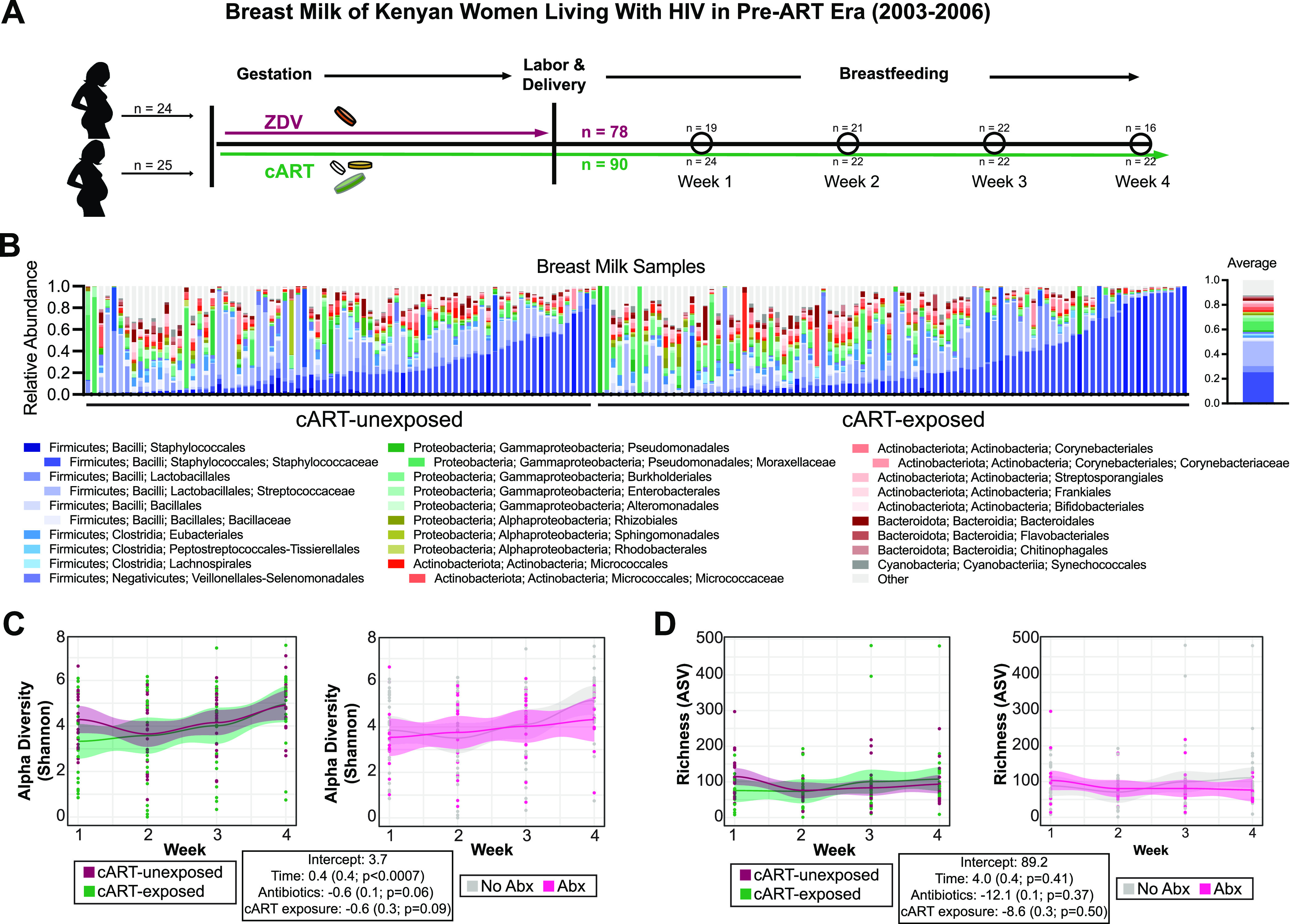
Bacterial microbiome analysis of breast milk samples over the first 4 weeks post-partum from WLHIV with or without cART exposure. (A) Overview of study design. (B) Individual and average relative abundance of bacteria at the Order and Family level in breast milk samples ordered by cART exposure and the most abundant increasing bacterial taxa (C) Loess plot of ASV alpha diversity against time points in cART-exposed or cART-unexposed samples (left) and women who used or did not use antibiotics (right). Statistical significance assessed by linear mixed effect model. (D) Loess plot of ASV richness against time points in cART-exposed or cART-unexposed samples (left) and women who used or did not use antibiotics (right). Statistical significance assessed by linear mixed effect model.

We performed bacterial microbiome 16S rRNA gene sequencing on available breast milk samples collected weekly during the first month postpartum from 25 women cART-exposed (*n* = 97 samples) and 24 cART-unexposed women (*n* = 83 samples). Seven samples from the cART-exposed group and five sample from the cART-unexposed group were dropped from the analysis due to insufficient read depth, resulting in 90 cART-exposed and 78 cART-unexposed breast milk samples (Fig. 1A). Forty-four out of 49 women had >2 samples included.

We found shared bacterial microbiota in breast milk from women regardless of cART-exposed group. The breast milk samples had high abundances of Staphylococcaceae (24% abundance ± 20%; present in 96% of breast milk samples), Streptococcaceae (20% abundance ± 19%; 95% of samples), Moraxellaceae (7% abundance ± 16%; 89% of samples), Micrococcaceae (2.7% abundance ± 5%; 91% of samples), Corynebacteriaceae (2.3% abundance ± 3%; 89% of samples), and Bacillaceae (2.1% abundance ± 4%; 82% of samples) (Fig. 1B). We found no differences in the abundance or prevalence of these core bacterial families due to time, cART or antibiotics use (Kruskal-Wallis; adjusted *P*-value range >0.09–0.99).

We used linear mixed effects models to determine if the diversity of the bacterial microbiome was changing over time, comparing women with and without cART exposure and controlling for antibiotic use and cART exposure. We found that alpha diversity significantly increased over time (*P* < 0.0007), in a similar manner in both cART-exposed and unexposed women, however cART exposure and antibiotics use were associated with a non-significant lower alpha diversity across time points (cART exposure *P* = 0.09; antibiotics use *P* = 0.06) (Fig. 1C). Richness did not significantly change over time, and median richness and changes in richness over time were not associated with cART exposure or antibiotic use (Fig. 1D).

**FIG 2:**
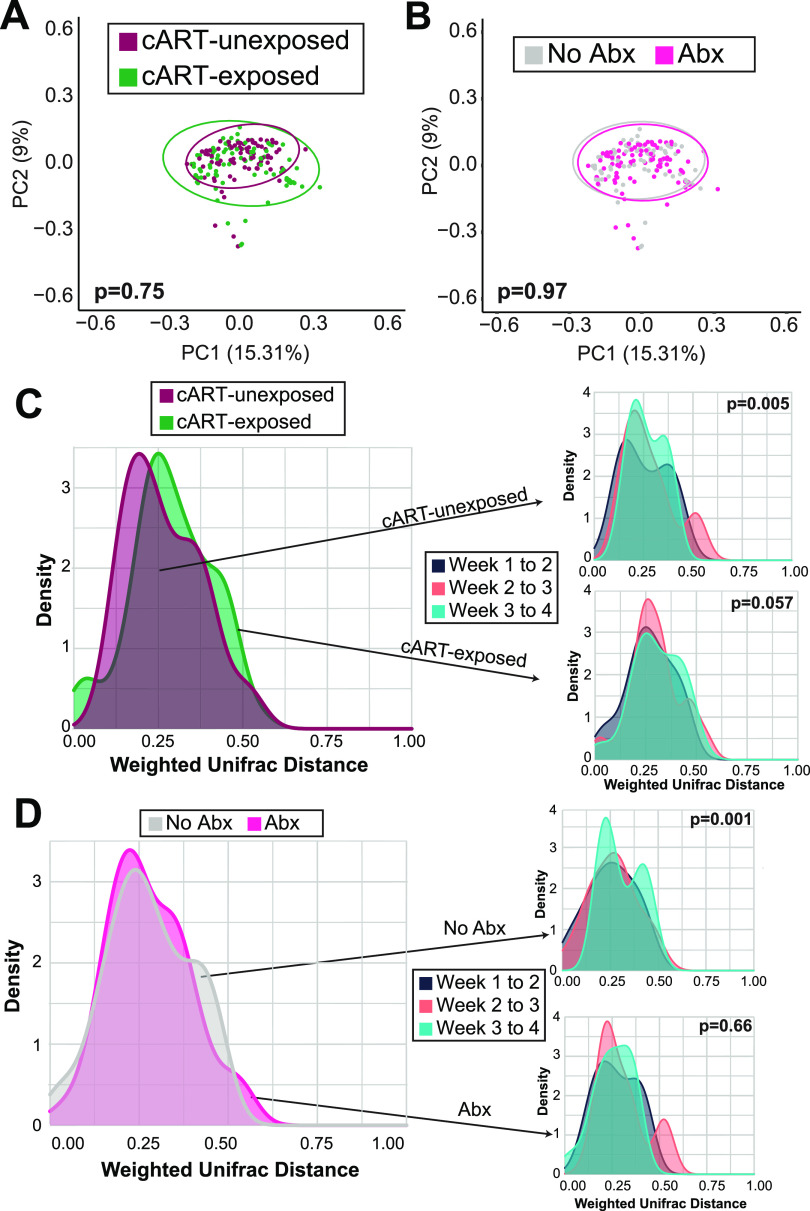
Bacterial microbiome beta diversity of breast milk samples over the first 4 weeks post-partum from WLHIV with or without cART exposure. (A) PCoA plot of ASV weighted UniFrac distances. Colors represent cART exposure. Statistical significance assessed by PERMANOVA. (B) PCoA plot of weighted UniFrac distances. Colors represent antibiotics or no antibiotics. Statistical significance assessed by PERMANOVA. (C) Density plot of weighted UniFrac, separated by cART exposure. Colors represent cART exposure (left) and weeks postpartum (right). Statistical significance assessed by PERMANOVA. (D) Density plot of weighted UniFrac, separated by antibiotics or no antibiotics. Colors represent cART exposure (left) and weeks postpartum (right). Statistical significance assessed by PERMANOVA.

We next determined whether breast milk bacterial microbiome beta diversity differed by cART exposure or antibiotic usage by performing PERMANOVA analyses with weighted UniFrac distances. We found that while there was no significant difference in breast milk bacterial microbiome beta diversity based on cART exposure or antibiotic use (cART-exposure *P* = 0.75; antibiotic use *P* = 0.97; Fig. 2A and B), there was a statistically significant difference due to time (*P* = 0.005; Fig. S1A in the supplemental material). In *post hoc* comparisons, statistically significant differences were detected between week 1 and week 4 (*P* = 0.006) and marginally statistically significant differences between week 2 and week 4, as well as week 3 and week 4 (*P* = 0.054). Moreover, bacterial microbiome beta diversity was marginally to significantly different when compared progressively across postpartum time regardless of cART exposure (cART-exposed *P* = 0.005, cART-unexposed *P* = 0.057; Fig. 2C). Although the beta diversity of women never using antibiotics was significantly different when compared progressively across postpartum time, it was not significant for women ever using antibiotics (never antibiotics use *P* = 0.001, ever antibiotics use *P* = 0.66; Fig. 2D). The change in beta diversity over time was only observed in the women never using antibiotics, which suggests the use of antibiotics can influence the breast milk bacterial microbiome.

**FIG 3:**
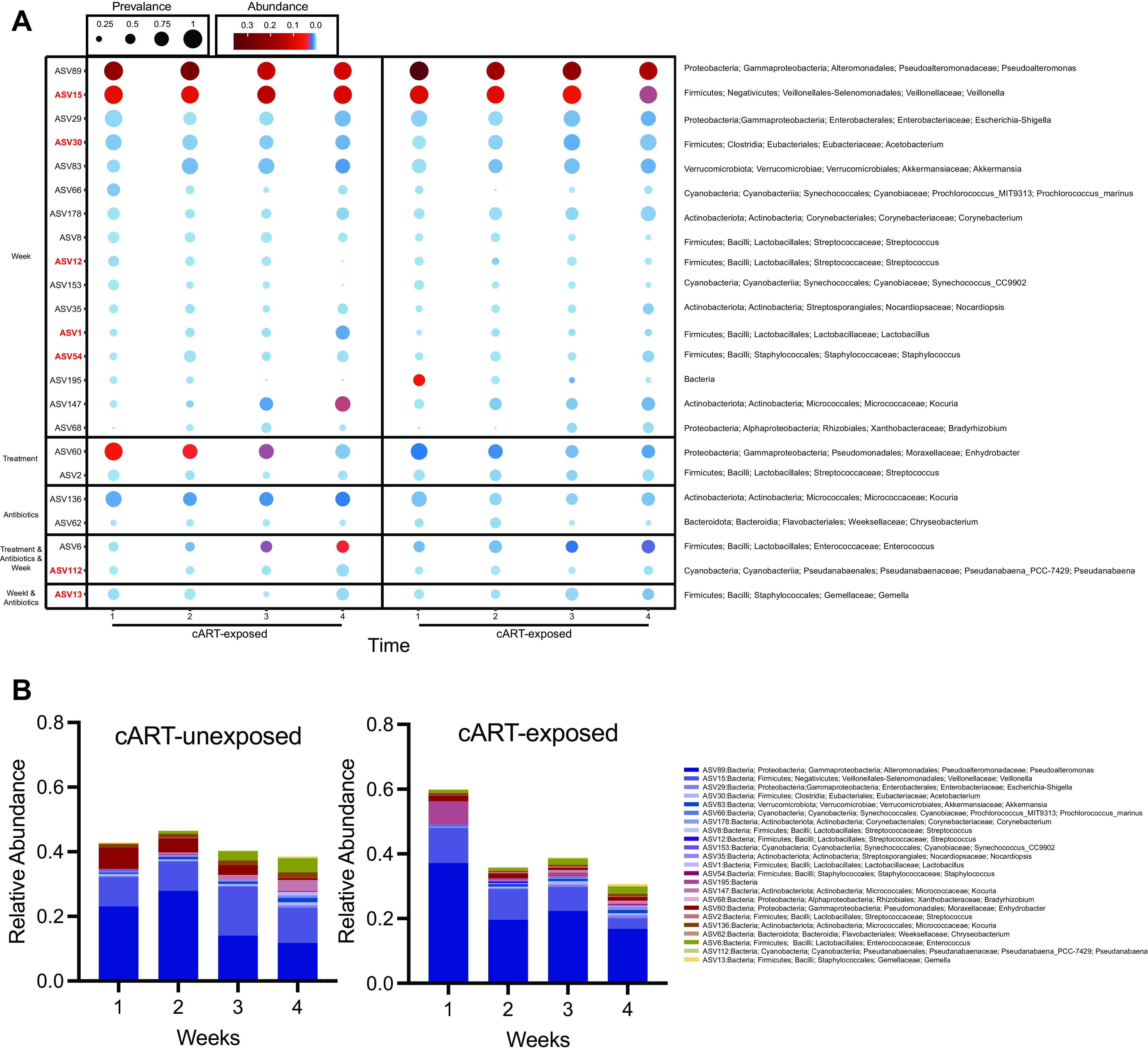
Discriminating analysis of bacterial ASVs by time, cART exposure or antibiotic uses. (A) Prevalence and abundance plot of candidate discriminating ASV determined by MaAsLin2. Size of the circle represents the prevalence and color represents the abundance of the ASV. (B) Relative abundance of the candidate ASVs by weeks postpartum.

Since our results indicate that the bacterial microbiome diversity of breast milk changes over postpartum time, we performed a multivariate analysis using MaAsLin2 (22). We identified 23 discriminating bacterial Amplicon sequence variants (ASVs) candidates that differed based on postpartum time, cART exposure or antibiotics usage (Fig. 3A and B, Fig. S1B). Of these, seven ASVs were significant after correcting for multiple comparisons: Staphylococcus, which decreased over time; Veillonella dispar, Acetobacterium, Lactobacillus, and Streptococcus which increased over time and Gemellaceae and Pseudanabaena, which were more abundant in cART-unexposed women (Fig. 3A–B). Taken together, these results suggest that postpartum time is the main factor predicting composition of the breast milk bacterial microbiome.

### Community states in breast milk bacterial microbiome.

**FIG 4:**
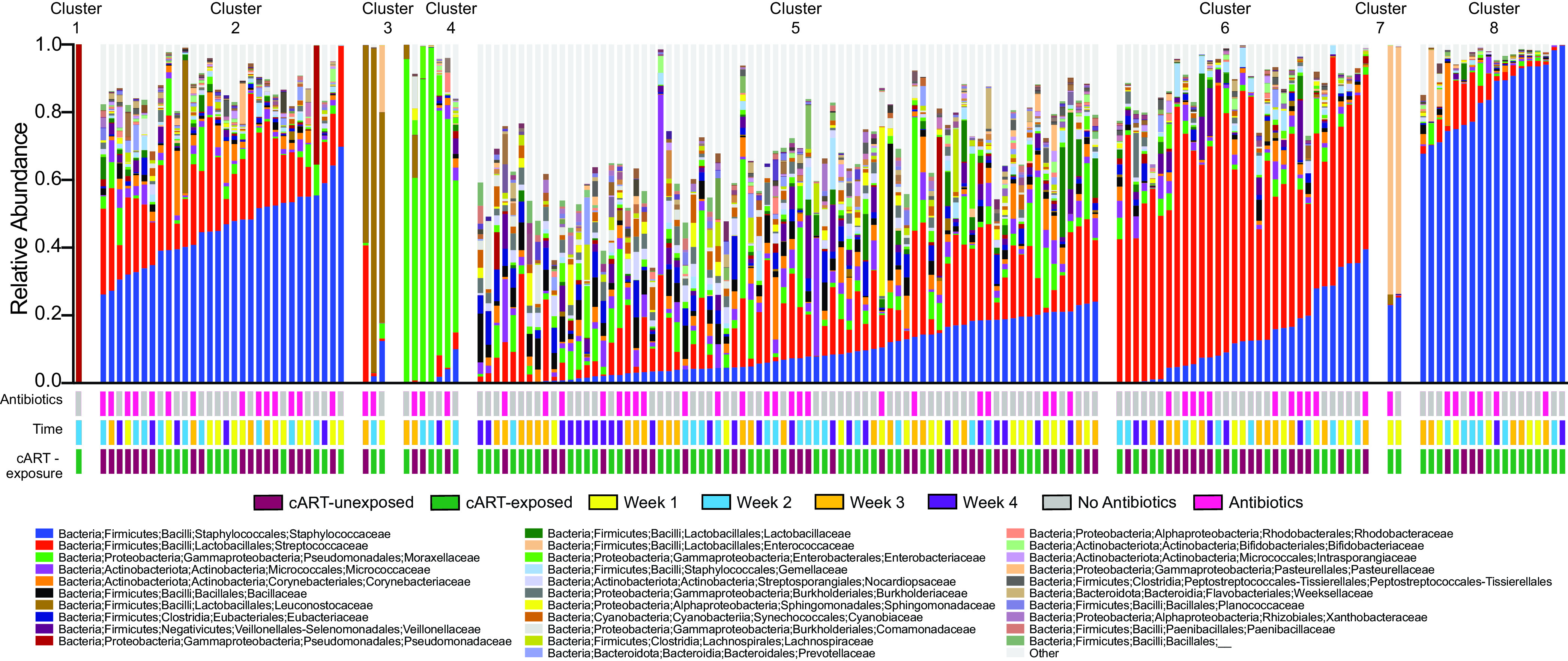
Breast milk bacterial community states. Relative abundance of bacteria at family level, clustered using k-means. Plot labeled with community state clusters.

Breast milk harbors a core bacterial microbiome that is shared across geographic regions but can also have diverse and unique bacterial profiles (5, 7). To determine the community state profiles in this Kenyan cohort, we used the k-means method to cluster the breast milk bacterial microbiome abundance at the family level. This resulted in eight distinct clusters indicative of bacterial community states (Fig. 4). Cluster 1 was dominant, with near 100% abundance, in *Pseudomonadaceae*. Clusters 2 and 8 were dominant in Staphylococcaceae with cluster 2 being 45% and cluster 8 being 84% abundant in Staphylococcaceae. Cluster 3 was dominant in Leuconostocaceae (72%), while cluster 4 was dominant in Moraxellaceae (76%). Cluster 5 was compromised of many taxa with the most abundant being Streptococcaceae (12%), Staphylococcaceae (9%), Moraxellaceae (6%), Micrococcaceae (4%), Bacillaceae (4%), Corynebacteriaceae (3%) and Eubacteriaceae (3%). Cluster 6 was dominant in Streptococcaceae (54%) and cluster 7 was dominant in Enterococcaceae (73%). Multinominal logistic regression indicated that community state clusters 5 and 2 were associated with postpartum time (q-value = 0.05). Specifically, 70% of the samples in community state cluster 2 were from weeks 1 and 2, while cluster 5 had 61% of samples from weeks 3 and 4 (Fig. S1C). These results corroborate our findings that breast milk bacterial microbiome alterations are associated with time postpartum.

## DISCUSSION

We investigated the bacterial microbiome in breast milk collected weekly over the first month postpartum from Kenyan WLHIV, comparing women that were and were not exposed to cART. In multivariate analyses that included postpartum time, cART exposure and antibiotic use, we found that time was the most significant factor in breast milk bacterial microbiome changes. Both alpha diversity and beta diversity changed significantly over time and beta diversity was also influenced by antibiotics usage. However, cART did not significantly impact the bacterial microbiome. We also found discriminating ASVs and community states associated with time. Taken together, our results indicate that the bacterial microbiome in the breast milk of WLHIV changes over time, independent of cART exposure.

Our study supports the resiliency of the breast milk bacterial microbiome, the core of which appears to be minimally altered by maternal HIV infection, immunosuppression or antiretroviral therapy, but malleable by direct selection pressure from antibiotics. Breast milk is highly dynamic, changing with the nutritional and immunologic needs of the infant. The colostrum and early milk are extremely cell-rich, with cell numbers declining rapidly over the first weeks of life and being largely without cells by 3–6 months (3, 23, 24). Protein content increases over time while fat and HMOs decrease (23, 25). Behavioral factors, such frequency of breastfeeding, and introduction of complementary foods further influence the qualities and quantity of the milk. The breast milk bacterial microbiome is undoubtedly influenced by these changes to the local mucosal immune environment and availability of substrates, which may explain both its resilience and why we observed changes in alpha diversity and beta diversity over time in our study. One previous study looked at changes over the first 12 months postpartum and found that breast milk alpha diversity did not change significantly, while beta diversity increased over the first 6 months and then decreased (3). Although our study also showed an increase in beta diversity, our results differed for alpha diversity, which could in part be due to differences in the time periods studied: the first 12 months postpartum in the previous study and 4 weeks post-partum in our study.

We found that the bacterial microbiome in the breast milk in our study had a common “core” bacterial taxon, comprising an array of bacterial families, with the most abundant and frequent families being Streptococcaceae and Staphylococcaceae. In addition, we also found eight distinct community state with unique compositions of bacteria. Most of the clusters had one dominant bacterial family, while only one of the community state clusters had different proportions of several abundant bacterial families. Therefore, although we find common core bacteria in these breast milk samples, these samples also have unique and diverse bacteria clusters. These findings are consistent with previous studies, in which although there are common shared bacteria, there are also differences between geographic regions as well as individuals (5, 7).

Our study has several strengths, including weekly breast milk collection. Using archived specimens from the era before cART was recommended and available to pregnant and postpartum mothers allowed us to have a comparison group of women who did not receive cART during breastfeeding, which would not be possible today. A limitation of our study is that our cART-unexposed group received ZDV antenatally. While ZDV has a very short half-life (1–2 h), we cannot exclude a potential effect of this drug on very early breast milk in the cART-unexposed group; this short-term antiretroviral drug exposure may explain the higher alpha diversity observed in the cART-unexposed group at week 1, when ZDV could still have an effect. Notably, there are some differences in contemporary cART regiment compared to those in this study period. For example, ZVD is not commonly used; non-nucleoside analogue reverse transcriptase inhibitors (NNRTIs), such as nevirapine, have also largely been replaced by integrase strand transfer inhibitors (INSTIs) (26). This study did not include HIV uninfected women and thus is limited to comparison between cART-exposed and cART-unexposed WLHIV. Additionally, one of the confounders present in the study was antibiotics usage, with more antibiotics being used in cART-unexposed women, although we did adjust for this factor in our models (Fig. S1D in the supplemental material).

In conclusion, we found the breast milk bacterial microbiome of Kenyan WLHIV changed dramatically during the first 4 weeks postpartum but was resilient to cART. Future studies are needed to compare breast milk bacterial microbiome in WLHIV on cART to HIV-uninfected women, as well as to analyze the interactions between bacterial microbiome and virome.

## MATERIALS AND METHODS

### Study population.

Two randomized clinal trials were independently conducted between 2003 and 2006 in the Mathare North Clinic in Nairobi, Kenya to compare the effects of different ART regimens, used for the prevention of mother to child transmission, on HIV levels in breast milk (Table 1) as previously described (19–21). Briefly, in the cART trial, women were excluded if their CD4 counts at enrollment were <200 or >500; there was no CD4 exclusion criteria in the ZDV trial. Pregnant WLHIV included here in this sub-study either received 300 mg of ZDV twice daily from 34 weeks until labor and every 3 h during labor until delivery but did not continue treatment postpartum; or received combination antiretroviral therapy of ZDV, nevirapine (NVP) and lamivudine (3TC) for 6 weeks prior to delivery and continued for 6 months postpartum. Breast milk was collected every 2–3 days for the first 4 weeks postpartum; the study presented here describes a longitudinal subset of these breast milk samples collected each week for the first 4 weeks postpartum. Twenty-four women that received the ZDV regimen contributed 83 longitudinal breast milk samples while 25 women that received cART (ZDV/NVP/3TC) contributed 97 samples.

### Bacterial microbiome 16S rRNA sequencing.

Breast milk samples collected in sterile containers were processed in Nairobi as previously described (19–21) and stored in liquid nitrogen. After the samples (500 μL) were thawed, 300 μL PBS was added, vortexed briefly and centrifuged at 20,000 × *g* for 10 min. The supernatant was removed, and DNA extraction was performed on the pellet using DNeasy PowerSoil kit (Qiagen). Negative controls consisting of PBS were processed in parallel to the breast milk samples to assess contamination during extraction, amplification and sequencing. Bacterial 16S rRNA sequencing was performed as previously described (27, 28). Briefly, PCR was performed on the DNA from the DNeasy eluate using primers for the V4 region (F515/R806). Libraries were sequenced on the Illumina MiSeq platform (v2, 2 × 250) at the ASU Biodesign Institute Genomics Core facility.

### Bacterial microbiome analysis.

Illumina sequencing reads (2 × 250 paired end reads) were processed through the QIIME2 using DADA2 to obtain denoised sequence reads and Amplicon Sequence Variants (ASV) (29). These ASVs were then classified with classify-sklearn algorithm using Silva database at 99% identity criterion for taxonomy (30). To remove contaminant ASVs, the Decontam (version 1.2.1) package in R, which uses the ‘prevalence’ method to identify contaminants, was applied at a threshold of 0.25 (isContaminant) (Fig. S2A in the supplemental material, top) (31). Contaminant ASVs at the intermediate threshold (0.25) and below (default = 0.1) were removed. In addition, one ASV, Burkholderia*-*Caballeronia*-*Paraburkholderia, present in strict threshold (0.5) and one ASV, Achromobacter, present in high abundance and similar proportions in both participant and PBS samples, but that was not called by decontam, were also removed (Fig. S2A, bottom). False positives ASVs with taxa such as Archaea, Mitochondrial, and Chloroplast were also removed from data analysis (Fig. S2B). The 180 breast milk samples had an average of 68,718 ± 86,910 reads (after contamination removal). To control for inter-sample depth variability, QIIME2 was used to rarefy data to 8,000 reads (subsampling without replacement) and create phylogenetic tree and taxonomy (Fig. S2C). Analyses were also performed using GreenGenes database (13_8) taxonomic classification that yielded similar results such as the change in diversities over time and the influence of antibiotics on the microbiome.

For 90 samples from cART-exposed and 78 samples from cART-unexposed women that had >8,000 reads, we performed core ecological metrics of alpha diversity, and weighted UniFrac distances of ASVs using QIIME2. To account for repeated measurements per person, we computed linear mixed-effects (LME) models, using R package nlme (version 3.1–148) (32), to compare changes in richness and alpha diversity across time points in cART-exposed and cART-unexposed patients. Antibiotic usage (at the time point) was also included in the LME models to account for possible confounding. PCoA plots were plotted using ggplot2 (version 3.3.1) in R (33) with Unifrac weighted distance. Weighted Unifrac distances were compared using vegan, permute and Adonis in R to compare changes in beta diversity across metadata using PERMANOVA (34). To assess changes over time and permute all time points within a participant, antibiotic usage was assigned to all time points if a participant was ever on antibiotics over the 4 weeks. We used strata parameter to account for repeated measurements per patients and margin parameter to account for marginal effect of each variable independently. Kruskal-Wallis with multiple correction was used to analyze the core bacterial microbiome for differences due to time, cART exposure and antibiotics. Furthermore, we used time, cART exposure, and antibiotic usage to identify differentiating ASVs using R package Microbiome Multivariable Association with Linear Models (MaAsLin2) (22). We used the default q-value threshold of 0.25 for significance.

### Community state analysis.

To obtain the community states present in the breast milk samples of this study we clustered our data with k-means method. We first used the R package factoextra (version 1.0.7) (35) to determine the optimal number of clusters and then used stats function k-means to cluster the relative abundance at family level into eight groups. To determine associations between community states and time, cART exposure or antibiotic use, we used R package mclogit (version 0.8.7.2) to perform multinomial logit models with random effects for patient ids (36); the Benjamini-Hochberg method was used to correct for multiple comparisons for the mclogit results.

### Data availability.

Sequence data have been deposited to the NCBI Sequence Read Archive under accession number PRJNA748003.
