# Supplementary material for: Dynamic Changes in Breast Milk Microbiome in the Early Postpartum Period of Kenyan Women Living with HIV Are Influenced by Antibiotics but Not Antiretrovirals
Source: Microbiol Spectr. 2022 Apr 6;10(2):e02080-21. doi: 10.1128/spectrum.02080-21 (PMC9045247; doi:10.1128/spectrum.02080-21)
Supplement: SUPPLEMENTAL FILE 1 — Supplemental material. Download SPECTRUM02080-21_Supp_1_seq8.pdf, PDF file, 0.8 MB [file spectrum02080-21_supp_1_seq8.pdf]

Supplementary Figure 1

A

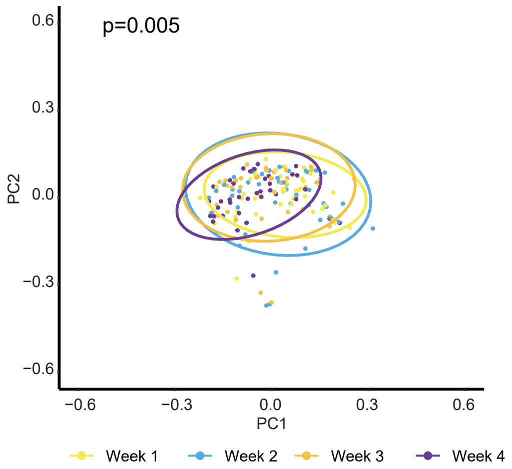

B

ASV	Taxonomy	Coef	Stderr	Pval	Qval	Metadata
**ASV1**	Staphylococcus	-403.123883	114.879230	0.000628	0.098008	Week
**ASV112**	Pseudanabaena	19.499809	6.475842	0.003015	0.204343	cART-unexposed
ASV112	Pseudanabaena	-17.654636	6.745308	0.009691	0.335960	Abx
ASV112	Pseudanabaena	-5.480874	2.448407	0.026531	0.404442	Week
**ASV12**	Streptococcus	74.849652	26.078619	0.004832	0.237637	Week
**ASV13**	Gemella	104.374818	33.792407	0.003170	0.204343	cART-unexposed
ASV13	Gemella	-68.534527	30.556663	0.026346	0.404442	Abx
ASV136	Kocuria	5.256168	2.219149	0.019534	0.400051	Abx
ASV147	Kocuria	4.049993	1.721538	0.019832	0.400051	Week
**ASV15**	Veillonella	46.679931	10.751032	0.000029	0.009162	Week
ASV153	Synechococcus	5.540040	2.156347	0.011317	0.353091	Week
ASV178	Corynebacterium	-1.540754	0.757435	0.043914	0.595706	Week
ASV195	Bacteria	-3.484685	1.489510	0.020515	0.400051	Week
ASV2	Streptococcus	401.654668	201.956488	0.051480	0.613400	cART-unexposed
ASV29	Escherichia-Shigella	12.615767	5.762699	0.031095	0.440989	Week
**ASV30**	Acetobacterium	20.423558	7.206390	0.005332	0.237637	Week
ASV35	Nocardiopsis	12.917974	5.291398	0.016023	0.384554	Week
**ASV54**	Lactobacillus	14.688113	4.903996	0.003275	0.204343	Week
ASV6	Enterococcus	-241.880764	120.392027	0.046166	0.600156	cART-unexposed
ASV6	Enterococcus	-90.561773	45.518201	0.048300	0.602790	Week
ASV60	Enhydrobacter	-19.213207	9.861383	0.053083	0.613400	cART-unexposed
ASV62	Chryseobacterium	34.368581	13.964519	0.014886	0.384554	Abx
ASV66	Prochlorococcus Marinus	8.114371	3.521431	0.022863	0.404442	Week
ASV68	Bradyrhizobium	7.324445	2.909140	0.012770	0.362208	Week
ASV8	Streptococcus	-81.199923	36.146982	0.026394	0.404442	Week
ASV83	Akkermansia	6.512734	2.916866	0.027222	0.404442	Week
ASV89	Pseudoalteromonas	5.876172	2.187649	0.007974	0.310980	Week

C

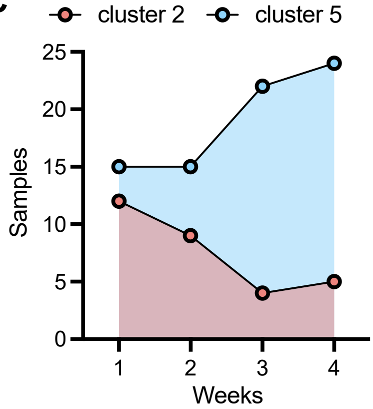

D

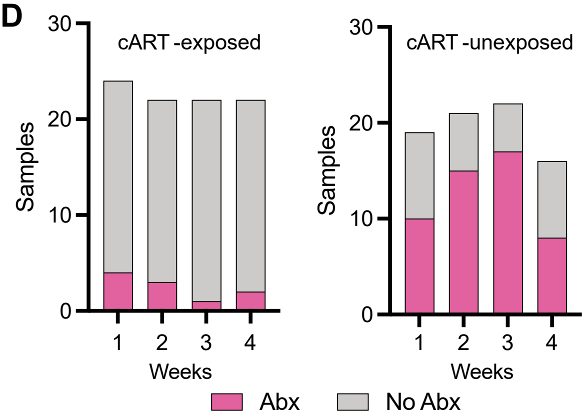

Supplementary Figure 1. Supplemental analysis of breast milk bacterial microbiome. (A) PCoA plot of weighted UniFrac distances. Colors represent weeks postpartum. Statistical significance assessed by PERMANOVA (B) Table of MaAsLin2output for candidate ASVs. Red font represents the ASVs that passed the multiple comparison correction. (C) Plot of the number of samples at each post-partum week in the community state groups. (D) Plot of samples from women who did or did not use antibiotics over the four weeks postpartum, stratified by cART exposure.

Supplementary Figure 2

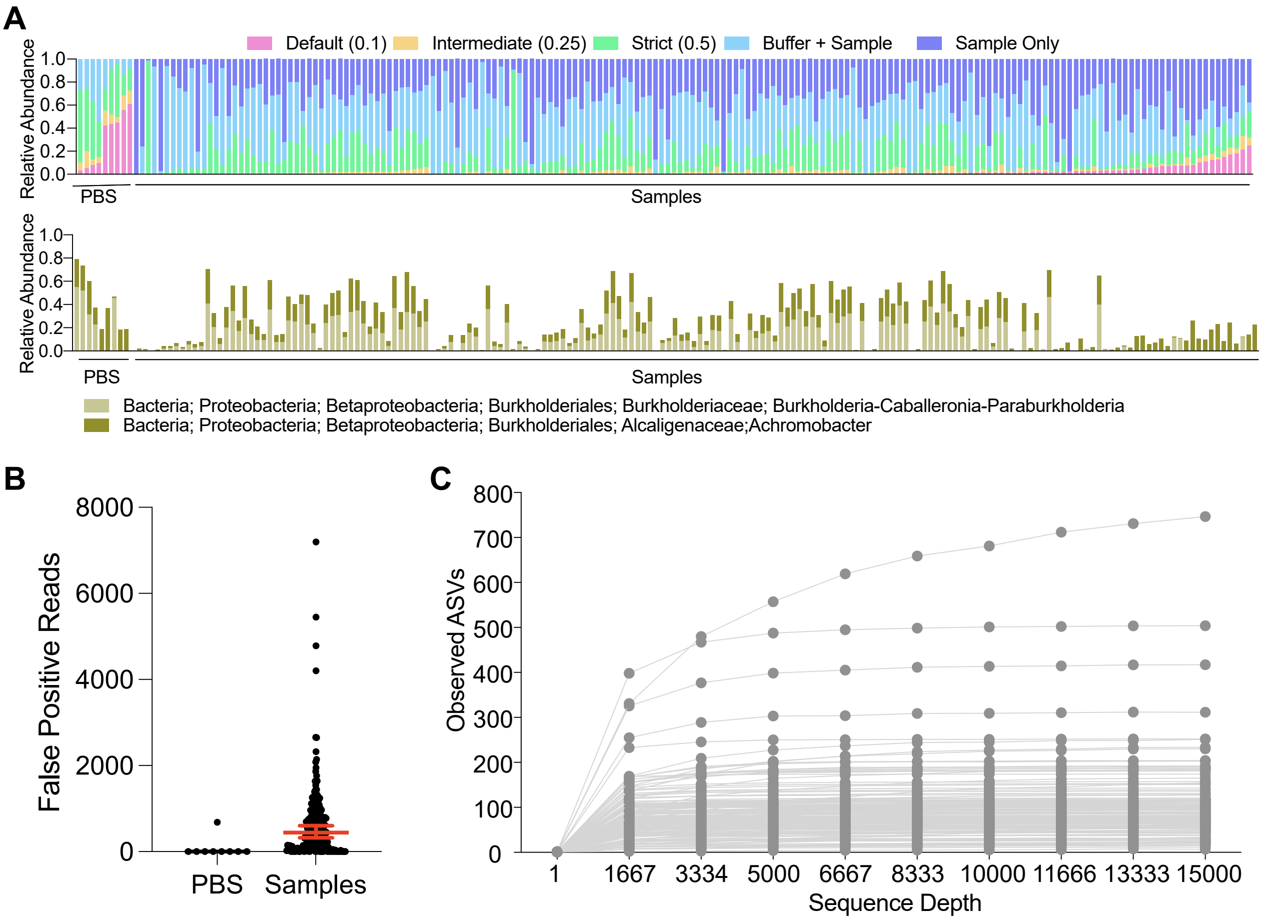

Supplementary Figure 2. Analysis of NGS dataset assessing for contamination sequences. (A) The top plot shows the relative abundance of 16S contaminants, as defined by prevalence thresholds from Decontam, for breastmilk samples and PBS buffer. The bottom plot shows the relative abundance of specific contaminate ASVs removed from the analysis due to high prevalence in both PBS and breastmilk samples. (B) Plot of number of false positive taxa removed from samples and PBS buffer controls. (C) Rarefaction plot of average bacterial ASV richness and alpha diversity at increasing read depth.
